# Change in Symptoms and Mucosal Findings after Proton Pump Inhibitor in Patients with Laryngopharyngeal Reflux

**DOI:** 10.1055/s-0044-1791642

**Published:** 2025-02-06

**Authors:** Min Woo Park, Sin Jae Kang, Jee Hye Wee

**Affiliations:** 1 Department of Otorhinolaryngology - Head and Neck Surgery, Kangdong Sacred Heart Hospital, Seoul, South Korea; 2 Department of Otorhinolaryngology - Head and Neck Surgery, Hallym University College of Medicine, Hallym University Sacred Heart Hospital, Anyang, South Korea

**Keywords:** laryngopharyngeal reflux, proton pump inhibitor, transnasal esophagoscopy

## Abstract

**Introduction** Although there are several reports of endoscopic findings of the larynx and esophagus in laryngopharyngeal reflux (LPR) patients, little is known about the correlation between change in symptoms and laryngeal and esophageal mucosal findings after proton pump inhibitor (PPI) treatment.

**Objective** The present study aimed to evaluate the changes in symptoms and mucosal findings of the larynx and esophagus using transnasal esophagoscopy (TNE) after PPI medication and to analyze their relationship in LPR patients.

**Methods** The current prospective study included 36 patients who complained of LPR symptoms. Reflux Symptom Index (RSI), Reflux Finding Score (RFS), and modified Los Angeles classification using TNE were obtained pretreatment and 8 weeks after treatment with PPIs.

**Results** Data from 22 patients who completed all examinations were analyzed. The mean age was 52.8 years, and 4 patients were men. The most common symptom was a globus sensation (54.6%). Both RSI ( *p <* 0.001) and RFS ( *p* < 0.001) were significantly improved after 8 weeks with PPI treatment. However, there was no correlation between improvement of RSI and RFS ( *p* = 0.350). Thirteen patients showed improvement in esophageal findings. However, there was no significant association between improvement of esophageal findings and RSI ( *p* = 0.350) or RFS ( *p* = 0.376).

**Conclusion** Although PPI treatment improved LPR symptoms and endoscopic findings, the change in symptoms was not related to endoscopic mucosal findings in the larynx and esophagus.

## Introduction

Laryngopharyngeal reflux (LPR) is one of the most common disorders in the head and neck region, defined as the retrograde flow of gastric contents into the larynx and pharynx. 1 It is likely to represent an extra-esophageal manifestation of gastroesophageal reflux disease (GERD). 2 For the diagnosis of LPR, several diagnostic tools, including esophagogastroduodenoscopy, barium swallowing test, and 24-hour double-probe pH monitoring, have been used. 1 3 However, empirical proton pump inhibitor (PPI) therapy has been commonly used based on symptoms and laryngoscopic findings, because these diagnostic tools are not practical to use in outpatient clinics. Recently, transnasal esophagoscopy (TNE) has become a wide-spread tool for evaluation of the esophagus, which is a simple, safe, and effective method that does not require sedation. 4 Transnasal esophagoscopy is a practical diagnostic procedure that can be easily performed in outpatient clinics to detect erosive esophagitis or gastric inlet patch in patients with LPR symptoms.

Proton pump inhibitors have been commonly prescribed for suspected LPR, because several studies report that LPR symptoms, laryngeal findings, and esophagitis improved after the use of PPIs. 5 6 7 8 9 10 However, there are few reports about the relationship between LPR symptoms and the change of endoscopic findings in the larynx and esophagus after PPI medication. Serial endoscopic examination is useful to demonstrate the association between LPR symptoms and endoscopic findings and to give diagnostic clues of LPR. Nevertheless, to the best of our knowledge, no longitudinal study of esophageal evaluation in LPR has been conducted yet.

In the present study, we investigated the change in LPR symptoms, laryngeal findings, and reflux esophagitis using serial TNEs after PPI treatment and analyzed their associations.

## Methods

### Study Design

The current prospective study was performed between October 2012 and March 2013, and it included patients with LPR symptoms. Approval for this study was obtained from our Institutional Review Board (no. 2014AN0070), and it was conducted according to the principles expressed in the Declaration of Helsinki. Written informed consent was obtained from all participants. Laryngopharyngeal reflux symptoms were defined as at least 1 symptom persisting for more than 3 months including hoarseness, throat clearing, throat pain, globus sensation, and cough that could not be explained by other etiologies. Exclusion criteria included age < 18 years, head and neck cancer, history of radiation therapy to the head and neck, prior voice pathology requiring therapy, and current use of PPI or H _2_ blocker. Of 40 eligible patients who met the criteria, 4 refused to participate in the current study. All patients underwent examinations, including symptom questionnaire, laryngoscopy, and TNE. Among 36 patients, 7 with Reflux Symptom Index (RSI) < 13 or Reflux Finding Score (RFS) < 7 were excluded. A total of 29 patients were prescribed PPIs medication (30 mg of lansoprazole or 20 mg rabeprazole) once a day for 8 weeks. All data were documented at pretreatment and 8 weeks after initiating treatment.

### Reflux Symptom Index

We recorded the severity of LPR symptoms using the RSI. The RSI proposed by Belafsky et al. 11 is a validated self-administered questionnaire that includes nine items. The questions are rated on a five-point Likert-type scale; higher values denote more severe impact on daily functioning. Based on normative data, an RSI score > 13 is clinically significant and highly suggestive of LPR.

### Reflux Finding Score

All patients underwent complete physical examinations and laryngeal endoscopy. The laryngoscopic findings were evaluated using 8 items, including subglottic edema, ventricular obliteration, erythema or hyperemia, vocal fold edema, diffuse laryngeal edema, posterior commissure hypertrophy, granuloma or granulation tissue, and endolaryngeal mucus. Reflux Finding Score, proposed by Belafsky et al., 12 ranged up to 26. The RFS has demonstrated high reproducibility and reliability, and a patient with scores > 7 points has 94% probability of presenting LPR.

### Transnasal Esophagoscopy

The examinations were performed with the 600-mm video endoscope (EE-1580K; Pentax, Tokyo, Japan) with 140° visual field, 5.1-mm tip diameter, and a 2.0-mm instrument channel while the patient was seated in a standard ear, nose, and throat (ENT) examining chair. Local anesthesia (xylocaine) in the form of nasal and oral spray was required. Reflux esophagitis were evaluated with modified Los Angeles (LA) classification 13 as follows: grade N: normal mucosa, grade M: minimal changes to the mucosa, such as erythema and/or whitish turbidity, grade A: nonconfluent mucosal breaks < 5 mm in length, grade B: nonconfluent mucosal breaks > 5 mm in length, grade C: confluent mucosal breaks < 75% circumferential, and grade D: confluent mucosal breaks > 75% circumferential. Dilation of the esophagogastric junction to twice the diameter of the endoscope in a reverse image of the cardia was diagnosed as esophageal hiatal hernia.

### Statistical Analysis

Chi-squared tests were used to compare the frequency distributions of categorical variables. For the entire group and each subgroup, comparisons between the pre and posttreatment variables were performed using Wilcoxon signed-rank test. Records were partitioned into subgroups depending on whether the RSI or RFS scores normalized after PPI treatment. The Fischer exact test was calculated to determine the probability that the association between symptoms and endoscopic findings. For all tests, a probability of < 0.05 was accepted as statistically significant. All statistical analyses were done with the IBM SPSS Statistics for Windows software, version 22.0 (IBM Corp., Armonk, NY, USA).

## Results

### Demographics

Data from 22 patients who completed all examinations were analyzed. The mean age was 52.8 ± 11.5 (range: 27–73) years. There were 4 men (18.2%) and 18 women (81.8%). Comorbidities included 6 patients with hypertension, 3 with osteoporosis, and 2 with diabetes mellitus. None of the patients reported to be smokers, but 3 patients were former smokers. The chief complaints were globus sensation in 12 patients (54.6%), throat pain in 7 (31.8%), voice problem in 2 (9.1%), and cough in 1 (4.5%).

### Comparison of Pre and Posttreatment Symptoms and Endoscopic Findings

**Fig. 1:**
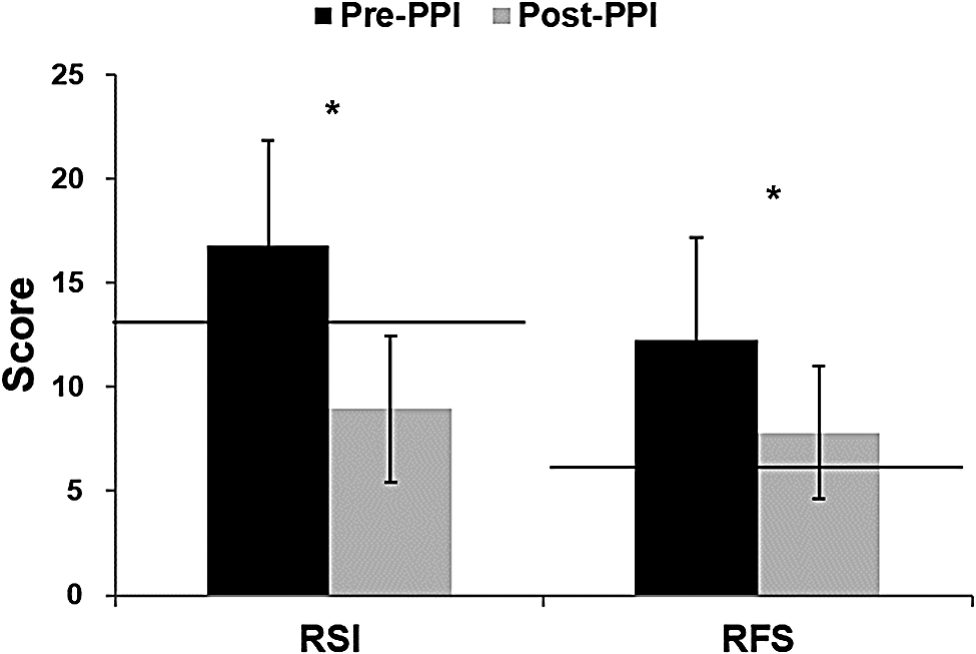
Pretreatment and posttreatment Reflux Symptom Index (RSI) and Reflux Finding Score (RFS) (mean ± standard deviation). Dashed lines represent normalization thresholds (RSI = 13 and RFS = 7). All RSI and RFS significantly decreased after proton pump inhibitor treatment. Note: * *p* < 0.05.

The comparison of the pre and posttreatment assessments is summarized in Table 1 . Hoarseness, throat clearing, postnasal drip, dysphagia, cough, globus sensation, and reflux symptoms improved significantly after PPI medication (all *p* < 0.05). Subglottic edema, vocal fold edema, ventricular obliteration, and thick endolaryngeal mucus decreased significantly after PPI medication (all *P* < 0.05). Pretreatment RSI and RFS were 16.77 ± 5.06 and 12.27 ± 3.53 respectively. After PPI medication, RSI and RFS improved significantly to 8.95 ± 4.93 ( *p* < 0.001) and 7.82 ± 3.17 ( *p* < 0.001), respectively ( Fig. 1 ).

**Table 1:** Changes in the Reflux Symptom Index and Reflux Finding Dcore after proton pump inhibitor treatment

Subitems	Pretreatment: mean ± SD	Posttreatment: mean ± SD	*p* -value
Hoarseness or voice problem	2.00 ± 1.41	1.14 ± 1.12	0.011
Clearing throat	3.23 ± 1.27	1.86 ± 1.39	< 0.001
Excess throat mucus or postnasal drip	1.73 ± 1.55	1.18 ± 1.37	0.042
Difficulty swallowing food, liquid, or pills	1.32 ± 0.94	0.41 ± 0.73	< 0.001
Coughing after eating or lying down	0.59 ± 1.10	0.23 ± 0.43	0.104
Breathing difficulties or choking episodes	1.00 ± 1.07	0.64 ± 0.79	0.201
Troublesome or annoying cough	1.36 ± 1.29	0.36 ± 0.66	< 0.001
Sensations of something sticking or lump in throat	3.59 ± 1.10	1.82 ± 1.43	< 0.001
Heartburn, chest pain, indigestion, or stomach acid coming up	1.95 ± 1.33	1.32 ± 0.94	0.019
*Total score*	16.77 ± 5.06	8.95 ± 4.93	< 0.001
Pseudosulcus (subglottic edema)	1.59 ± 0.80	1.00 ± 1.02	0.006
Vocal fold edema	1.64 ± 0.73	1.14 ± 0.35	0.002
Ventricular obliteration	2.00 ± 1.19	0.91 ± 1.34	0.001
Diffuse laryngeal edema	1.36 ± 0.49	1.14 ± 0.77	0.135
Erythema/hyperemia	2.00 ± 0.5	1.2 ± 0.8	0.131
Posterior commissure hypertrophy	1.91 ± 0.68	1.59 ± 0.80	0.050
Thick endolaryngeal mucus	1.59 ± 0.80	0.64 ± 0.95	0.002
Granuloma/granulation tissue	0.18 ± 0.60	0.00 ± 0.00	0.162
*Total score*	12.27 ± 3.53	7.82 ± 3.17	< 0.001

Abbreviation: SD, standard deviation.

Esophageal finding by TNE revealed grade B in 2 patients, grade A in 9 patients, and grade M in 9 patients. Two patients showed grade N, and one patient showed hiatal hernia. No patients showed grade C or D. Esophageal finding after 8 weeks of PPI medication showed grade A in 3 patients, grade M in 14 patients and grade N in 5 patients. The case of hiatal hernia remained unchanged.

### Relationship Between Reflux Symptoms and Endoscopic Mucosal Findings

The frequencies of RSI and RFS normalization are shown in Table 2 . Following PPI treatment, the RSI normalized for 15 of the 22 (68.2%) patients. However, the RFS did not normalize for 15 (68.2%) patients. Both RSI and RFS normalized in only 6 (27.3%) patients. There was no significant correlation between improvement of the RSI and RFS ( *p* = 0.350).

**Table 2:** Frequencies of normalization of the Reflux Symptom Index and Reflux Finding Score following proton pump inhibitor treatment

		Reflux Finding Score		
		Normalized	Not normalized	Total
**Reflux Symptom Index**	**Normalized**	6	1	7
	**Not normalized**	9	6	15
	**Total**	15	7	22

Both patients showing grade-B erosive esophagitis improved to grade A. Of the 9 patients with grade A reflux esophagitis, all except one improved to grade M. Although 3 patients showing grade M improved to grade N, other 6 patients with grade M did not show improvement. There was no significant association between improvement of the RSI and modified LA classification ( *p* = 0.376), or the RFS and modified LA classification ( *p* = 0.648) ( Table 3 ).

**Table 3:** Associations involving erosive esophagitis and the Reflux Symptom Index and Reflux Finding Score following proton pump inhibitor treatment

		Erosive esophagitis		
		Improvement	No change	Total
**Reflux Symptom Index**	**Normalized**	10	5	15
	**Not normalized**	3	4	7
**Reflux Finding Score**	**Normalized**	5	2	7
	**Not normalized**	8	7	15
	**Total**	13	9	22

## Discussion

The present prospective study found no significant association between the symptoms and TNE laryngeal or esophageal findings, although the RSI and RFS significantly improved after treatment with PPIs.

Laryngopharyngeal reflux can be explained as the retrograde flow of gastric contents into the larynx and pharynx.

Laryngopharyngeal reflux is thought to induce various laryngopharyngeal symptoms due to direct mucosal injury of the laryngopharynx by gastric contents or vagally mediated reflexes due to acidification of the distal esophagus. 14 The laryngeal tissue certainly lacks the protective mechanisms of the esophagus and is highly vulnerable to any exposure to acid, pepsin, or bile acids. 15

There is no standard method for diagnosing LPR. In the past, ambulatory 24-hour pH monitoring was considered the gold standard for diagnosing reflux, but the test has a difficult access and poor adherence due to the inconvenience suffered during the procedure. Furthermore, recent studies have shown it to be unreliable in patients with laryngeal symptoms. 3 Laryngopharyngeal symptoms or endoscopic findings of the larynx and esophagus are frequently used to diagnose LPR. 16 Throat clearing, persistent cough, globus sensation, and voice changes are the most frequent symptoms of LPR. Although the symptoms related to LPR are confused with those due to other causes such as vocal fold lesions, laryngeal tumors, and infectious diseases of the nasal cavity or pharynx, RSI is a self-administered questionnaire with excellent validity and reproducibility, based on frequent symptoms in LPR patients. 11 Laryngoscopy is the most frequently used test for evaluation of the laryngeal mucosa. It is a simple and safe instrument to detect direct mucosal changes of the larynx and pharynx, although it is nonspecific for establishing reflux. The RFS is a validated scoring system for evaluating the changes of laryngoscopic findings related to LPR. 12

The endoscopic assessment of esophageal mucosal changes in patients with reflux symptoms is important to make accurate treatment decisions and assess its severity. Several studies have reported that endoscopic abnormalities in the esophagus were used to give a diagnosis of LPR in the patient with LPR symptoms. The LA classification system is the most widely used validated system to describe the endoscopic appearance of reflux esophagitis and grade its severity. 17 However, we used a modified LA classification with the added grades M and N, because minimal changes are considered as one of the endoscopic findings of reflux esophagitis in the Asian population. 18

Esophagoscopy has been used to examine the presence of erosive esophagitis, suggesting GERD as the underlying etiology, to detect heterotopic gastric mucosal patches of the proximal esophagus, and to reassure patients after excluding important esophageal causes. 19 Recently, esophageal evaluation using TNE has become widely practiced. It is a safe, efficient, and unsedated office-based procedure with diagnostic capabilities equal to sedated conventional esophagoscopy. 4 In this study, we examined the esophageal findings using TNE in the office without the need for sedation.

Validated tools are required to assess various symptoms and mucosal findings before and after treatment of LPR, so we assessed patients' symptoms as well as laryngeal and esophageal findings using the RSI, RFS, and modified LA classification.

Patients with reflux symptoms usually undergo empirical PPI treatment because of the difficulty in differentiating the reflux of acid from gastric juice. Although empirical PPI treatment is efficient in patients with suspected LPR, the true effect of PPI treatment on LPR is controversial. 20 In some prospective studies with a placebo control, PPI showed marked improvement in reflux symptoms and signs. However, other studies failed to demonstrate significant improvement when compared to placebo. 21 Our results showed a similar result, in that PPI treatment resulted in a notable improvement of reflux symptoms and endoscopic findings of the larynx and esophagus, although improvement of the esophagus was not significant.

Our study showed no significant association between change in symptoms and laryngeal and esophageal findings after PPI treatment. This is in accordance with previously published data demonstrating a poor correlation between laryngeal exam with symptom severity and symptom improvement over time. 20 22 Other studies also reported that laryngoscopic findings are not a reliable test for the evaluation of response to therapy, and no significant relationship between LPR and endoscopic esophagitis was found. 23 The possible reason why our result showed no correlation between symptoms and endoscopic findings of the larynx and esophagus is either that endoscopy has low power for detection of mucosal abnormality, 24 there is not enough time to show mucosal changes after initiating PPI medication, 10 or there are multiple factors affecting reflux symptoms. 25 A systematic review reported that the treatment success includes a placebo effect, which has been demonstrated to play a very significant role in LPR. 21

The limitations of the current study are as follows: this study has a relatively small number of cases, and the follow-up period is short. We did not identify reflux using dual pH monitoring, so we do not know where the reflux was present and if it led to laryngopharyngeal symptoms or not. Further studies are needed for the evaluation of mucosal changes in a larger sample with longer follow-up duration.

## Conclusion

Proton pump inhibitor medication may improve the reflux symptoms and mucosal findings of the larynx and esophagus in patients with LPR symptoms. However, the improvement of reflux symptoms is not correlated with improvements of endoscopic findings of the larynx and esophagus.
